# A COVID-19 Emergency Response for Remote Control of a Dialysis Machine with Mobile HRI

**DOI:** 10.3389/frobt.2021.612855

**Published:** 2021-05-07

**Authors:** Hassam Khan Wazir, Christian Lourido, Sonia Mary Chacko, Vikram Kapila

**Affiliations:** Mechatronics, Controls, and Robotics Laboratory, Mechanical and Aerospace Engineering Department, NYU Tandon School of Engineering, Brooklyn, NY, United States

**Keywords:** COVID-19, interface, human-robot interaction, manipulation, remote interaction, robotics

## Abstract

Healthcare workers face a high risk of contagion during a pandemic due to their close proximity to patients. The situation is further exacerbated in the case of a shortage of personal protective equipment that can increase the risk of exposure for the healthcare workers and even non-pandemic related patients, such as those on dialysis. In this study, we propose an emergency, non-invasive remote monitoring and control response system to retrofit dialysis machines with robotic manipulators for safely supporting the treatment of patients with acute kidney disease. Specifically, as a proof-of-concept, we mock-up the touchscreen instrument control panel of a dialysis machine and live-stream it to a remote user’s tablet computer device. Then, the user performs touch-based interactions on the tablet device to send commands to the robot to manipulate the instrument controls on the touchscreen of the dialysis machine. To evaluate the performance of the proposed system, we conduct an accuracy test. Moreover, we perform qualitative user studies using two modes of interaction with the designed system to measure the user task load and system usability and to obtain user feedback. The two modes of interaction included a touch-based interaction using a tablet device and a click-based interaction using a computer. The results indicate no statistically significant difference in the relatively low task load experienced by the users for both modes of interaction. Moreover, the system usability survey results reveal no statistically significant difference in the user experience for both modes of interaction except that users experienced a more consistent performance with the click-based interaction *vs.* the touch-based interaction. Based on the user feedback, we suggest an improvement to the proposed system and illustrate an implementation that corrects the distorted perception of the instrumentation control panel live-stream for a better and consistent user experience.

## 1 Introduction

Last few decades have witnessed widespread adoption of robotic solutions by several industries for operations that are considered difficult or dangerous for humans to perform (Trevelyan et al., 2008). In the automotive industry, for example, heavy-duty industrial manipulators form an integral part of the assembly line (Hägele et al., 2016) and one would be hard-pressed to find an automotive manufacturing facility that does not employ some sort of robotic assistance. Moreover, robots are actively being developed, examined, and used for inspection, decontamination, and decommissioning of nuclear plants (Nagatani et al., 2013; Krotkov et al., 2017); search and rescue operations following natural, industrial, and man-made disasters (Murphy et al., 2008); and exploration in outer space (Yoshida and Wilcox, 2008). The above examples have one common thread, i.e., obviating the exposure to harm and risk to human safety. Thus, when operating in hazardous environments, in most cases the robots act as a physical extension of their human operators to enhance their dexterity, sensory experience, and cognition (Wang et al., 2015). Endowing a human operator with the ability to utilize the robot to its maximum potential requires the development of intuitive user interfaces for human-robot interaction (HRI). In recent years, several advancements have been made to render the HRI as seamless as it can be.

HRI is a rapidly advancing research field with several active areas of application that include human supervised control of robots, autonomous robot control, and human-robot social interaction (Sheridan, 2016). The human supervised control can be further divided into proximal *vs.* remote control, the latter of which includes teleoperation and telerobotics (Sheridan, 1992). In a hazard-prone, high-risk environment, the use of remotely controlled robots is preferable over proximally controlled robots because the human operator can perform the required tasks from a safe remote location. Varied HRI modalities for telerobotics have been developed over the years and each approach achieves a particular objective. Some early examples of HRI for telerobotics include using a joystick for teleoperation (Yamada et al., 2009), performing stroke gestures on a touchscreen (Sakamoto et al., 2008) and pointing gestures using a camera (Abidi et al., 2013), and using a wearable sleeve (Wolf et al., 2013). In recent years, as mobile devices (e.g., smartphones and tablets) have become ubiquitous in our personal and work environments, users have gained increased comfort in utilizing the rear-facing cameras of mobile devices to interact with their environments. Since mobile devices with well-endowed sensing, interaction, communication, and computing functionality are readily available to the common user, mobile mixed-reality interfaces have become greatly accessible and do not require research-grade devices to implement algorithms that were previously thought to be computationally expensive. Recent implementations of augmented reality (AR) based approaches include tracking a single or multiple fiducial markers on the robot (Hashimoto et al., 2011; Kasahara et al., 2013) or its surroundings (Frank et al., 2017a; Chacko and Kapila, 2019) to determine the pose of the robot or objects in its workspace. Other studies have used this approach for multi-robot tracking and control (Frank et al., 2017b). Although marker-based tracking has its merits, with the advent of markerless technologies, e.g., Google’s AR Core (Lanham, 2018; Google, 2020), the tracking can be performed in even unstructured environments while using highly intuitive user interfaces. Studies such as Frank et al. (2017a) and Chacko and Kapila (2019) have explored the potential of directing a robot manipulator to perform pick-and-place tasks using virtual elements in a semi-autonomous manner with the aid of a human collaborator. Another study suggests the use of virtual waypoints to guide a robot along a path (Chacko et al., 2020). With telerobotics and HRI being used for myriad applications, we propose to use these approaches in a healthcare setting and show that telerobotics and intuitive HRI can obviate the need for patients and healthcare workers to be exposed to high-risk interactions during a pandemic.

Medical caregivers such as doctors and nurses share physical space and interact with patients routinely. These shared spaces have a higher concentration of pathogens, which makes their occupants particularly susceptible to contracting bacterial and viral infections. The situation is exacerbated in the case of an epidemic, or more importantly a pandemic, which can lead to a widespread shortage of personal protective equipment (PPE) and increase the risk of contagion for both the caregivers and patients in a medical facility. A contemporary, and still developing, example of this situation is the spread of the novel coronavirus pandemic across the world, including in the United States. Since the spring of 2020, there has been a massive global shortage of PPE, including face masks, eye protection, respirators, gloves, and gowns (Ranney et al., 2020). This PPE shortage has been a major barrier in responding effectively to the pandemic and in mitigating the resulting spread of Coronavirus Disease 2019 (COVID-19). Essential healthcare workers, such as first responders, nurses, and doctors have been forced to forgo or reuse PPE when working with patients with or without COVID-19 to preserve their limited stocks. Additionally, the novel coronavirus has been found to transmit asymptomatically, i.e., through infected patients who do not yet display any symptoms (Mizumoto et al., 2020), at a significant rate, thus markedly increasing the likelihood of cross-contamination during the treatment and care of all patients. Healthcare workers are additionally exposed to the risk of infection through interaction and contact with fomites, including medical devices or instrument panels, and subsequently transmitting the disease to coworkers (Klompas et al., 2020). Many healthcare providers caring for COVID-19 patients have become infected and even lost their lives due to a lack of sufficient access to PPE (Wang et al., 2020). In addition to increasing the strain on an already overloaded healthcare system, such a lack of protection poses a significant threat to the morale of healthcare workers and their families.

**FIGURE 1:**
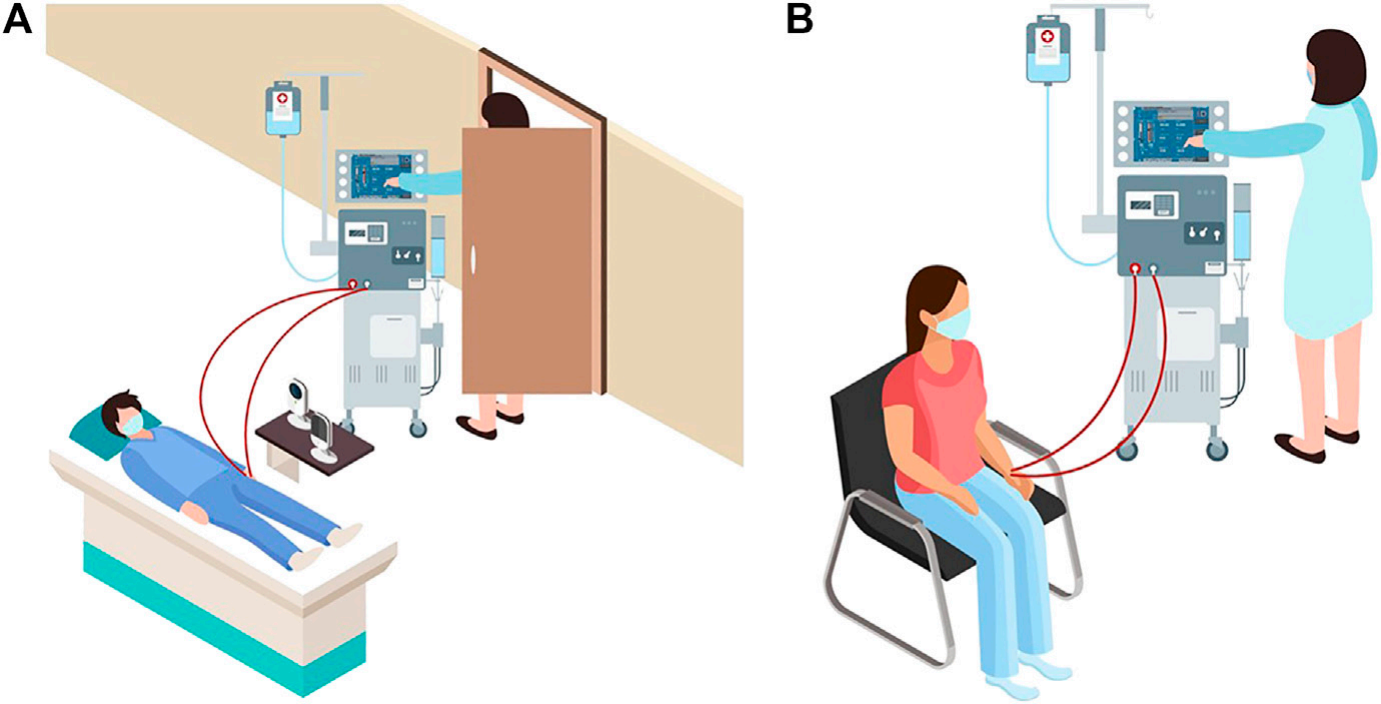
**(A)** Schematic representation of a dialysis patient receiving treatment at a hospital during the COVID-19 pandemic. The image shows use of baby monitors and reluctance of healthcare workers in entering the patient room. **(B)** A typical schematic representation of a patient receiving treatment at a dialysis center.

With the shortage of PPE, patients without COVID-19 who need critical and/or life-saving treatments also face increased risk in healthcare facilities (Naicker et al., 2020), including patients on dialysis. Such patients tend to be severely immunocompromised and are at a high risk of suffering serious complications if infected by the virus, as reported in China (Naicker et al., 2020). To minimize the risk of cross-contamination and infection, hospitals and dialysis centers have implemented strict protocols with multiple additional precautions in dialysis units for staff members, patients, and their family members (Naicker et al., 2020). However, dialysis centers have been plagued by staff, equipment, and PPE shortages. In fact, at the peak of the COVID-19 pandemic in New York City, a headline in the city’s paper of record *The New York Times* declared that “Dialysis Patients Face Close-Up Risk From Coronavirus,” (Abelson, 2020). During this period, healthcare workers sought to minimize visits with dialysis patients by using baby monitors and performing physical interaction with dialysis machines without fully entering the patient rooms (see Figure 1A). To mitigate the plight of these patients and avoid healthcare worker exposure, concerned authorities, such as the Food and Drug Administration (FDA), have encouraged expanding the non-invasive remote monitoring of such patients (FDA, 2020). To remotely determine whether a specific patient requires help, many healthcare device manufacturers are rolling out Internet of Things (IoT) devices to remotely monitor bio-signals relating to their temperature, heart rates, respiration rates, etc., (Hale, 2020). These remote systems are important tools for avoiding the overcrowding of emergency rooms and hospitals and reducing the unnecessary exposure of vulnerable people to pathogens. Historically, most of the research around medical robotics has concentrated on surgical teleoperation robots such as the DaVinci robot (Intuitive Surgical, Mountain View, CA), and is more focused toward patient safety during surgical procedures by mitigating human error and promoting minimally invasive procedures. Other medical robotic approaches focus on augmenting the doctor’s vision with virtual overlays to provide additional information (Liao et al., 2010; Wang et al., 2014). Some social and companion robots are available that target the elderly (Wada et al., 2005) or serve as emotional support (Logan et al., 2019), however there is a dearth of examples of telerobots that can be used to manipulate medical devices using intuitive HRI. There are autonomous robots that can deliver medications throughout hospitals (Murai et al., 2012), and a study explored the development of a tele-nursing robot (Li et al., 2017) that can navigate and interact with objects in the environment, but these solutions are either not relevant to this study or are cost prohibitive to be rapidly deployed in case of a pandemic.

**FIGURE 2:**
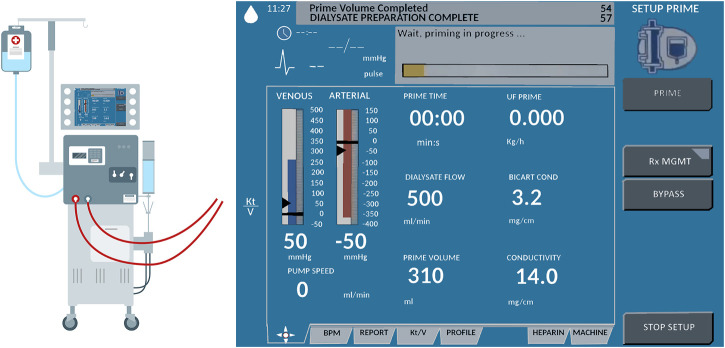
Schematic representation of Gambro X-36 Phoenix dialysis machine and its touchscreen interface.

In this paper, we propose to create an emergency, non-invasive remote monitoring and control response system that addresses the needs of a highly vulnerable population: patients with severe kidney diseases. A viable solution for remotely monitoring and controlling a dialysis machine’s instrumentation panel poses several design challenges. Typically, dialysis centers consist of multiple reclining chairs or beds with attendant dialysis machines placed next to them (see Figure 1B). Potential solutions for remotely manipulating the dialysis machine’s instrument panel include: (1) accessing embedded firmware of medical devices and (2) retrofitting the machine with a teleoperated robotic manipulator. As medical devices are sensitive instruments with proprietary firmware, varied software architectures, and individualized system requirements, it is not feasible to create a generalized framework to access the embedded firmware for remotely monitoring and controlling different medical instruments using smartphone/tablet-based third-party apps, especially as expeditiously as a pandemic emergency demands. Thus, retrofitting dialysis machines with teleoperated robotic arms, which can be easily mounted or removed as needed, is deemed as the most viable option. We envision a remote-monitoring-and-control framework wherein a camera-equipped robotic manipulator interacts with the instrument control panel of the dialysis machine, thus reducing the risk of COVID-19 exposure for both patients and healthcare providers. Our proposed solution can address the shortage of PPE in the heathcare facilities during a pandemic, enabling patients who require dialysis to continue receiving the life-saving treatment in isolation. At the same time, staff members in dialysis units can continue to provide high quality care with a relatively low risk of cross-contamination. This work’s engineering merits involve piloting a framework to quickly retrofit available dialysis machines with robust off-the-shelf four degrees-of-freedom (DoF) robotic manipulators and supporting remote management of the device instrumentation panel with high fidelity. Thus, in the proof-of-concept study of this paper, we recreate and live-stream the instrument control panel touchscreen (ICPT) of a commonly used dialysis machine, the Gambro X-36 Phoenix (Baxter International Inc., Deerfield, IL) (see Figure 2), to replicate and access it on a remote user’s tablet computer touchscreen (TCT). Moreover, we develop the control framework for the robot manipulator to achieve precise and accurate remote manipulation of the dialysis machine’s ICPT. We test our intuitive smartphone/tablet-based interface with over 30 users. Our future work will investigate wider applications of this framework to diverse medical instruments in the post COVID-19 era.

The paper is organized as follows. Section 2 elaborates on the materials and methods used in the study. This section provides details on the design of the robot manipulator and the user interface, the development of the communication architecture and marker detection, and the robot operation. Section 3 explains the system evaluation metrics used in this study. These metrics include a quantitative study about the accuracy of the robot and the user interaction, as well as qualitative studies about the user experience while operating the robot remotely. Following this, the results of system evaluation are provided and discussed in Section 4 and an improvement is suggested to render a distortion-free perception of the ICPT on the user TCT. Finally, Section 5 provides concluding statements and discusses the future direction of the research.

## 2 Materials and Methods

**FIGURE 3:**
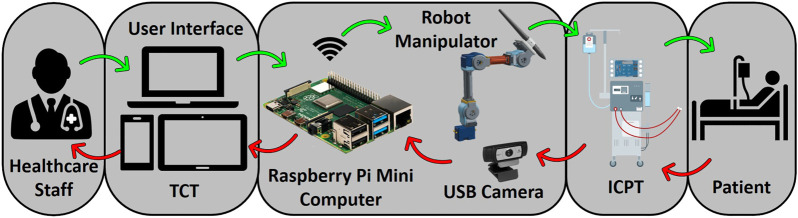
Schematic of a remote monitoring and control system for medical instruments. A healthcare worker interacts with the video-feed from a camera on a user interface (UI) hosted on a tablet computer touchscreen (TCT). The user commands are processed to control a robot manipulator to interact with the instrument control panel touchscreen (ICPT) of a dialysis machine serving a patient.

The method proposed in this paper uses an off-the-shelf four DoF robotic manipulator equipped with a USB camera. The robot base and camera stand are fixed on a board, making the system installation and operation simple, just requiring the user to properly locate the robot in front of its workspace and point the camera to a touchscreen (representing a dialysis machine ICPT) with which the robot manipulator is required to interact. The HRI user interface (UI) consisting of a mobile application (App) is connected to the same wireless network as the robot manipulator system. To identify the surface plane of action of the robot, the mobile App uses the camera’s video-feed which includes a 2D image marker located in the plane of the ICPT, in front of the robot manipulator. The mobile App determines this plane of action (i.e., robot workspace) based on the dimensions of the robot and its *computed* position relative to the image marker. With the mobile App executing on a hand-held smartphone or tablet, when a user taps on the TCT at any location of the displayed surface of operation, an algorithm transforms the tapped location’s pixel coordinates to a corresponding location coordinate in the workspace and frame of reference of the robot and sends it to the robot manipulator controller. Given this *commanded* position, another algorithm on the robot manipulator controller uses inverse kinematics to calculate a set of joint angles that can be used to attain the given position and orientation of the robot end effector and provides a solution to reach the specified location in space (Craig, 2018). Then, in a sequence of steps, the system plans a path, moves the robot manipulator to go to the desired location on the ICPT, taps on the desired location, and returns to its home position to wait for the next instruction. Figure 3 illustrates the components and interconnections of the proposed dialysis machine HRI environment.

### 2.1 Robot Hardware

**FIGURE 4:**
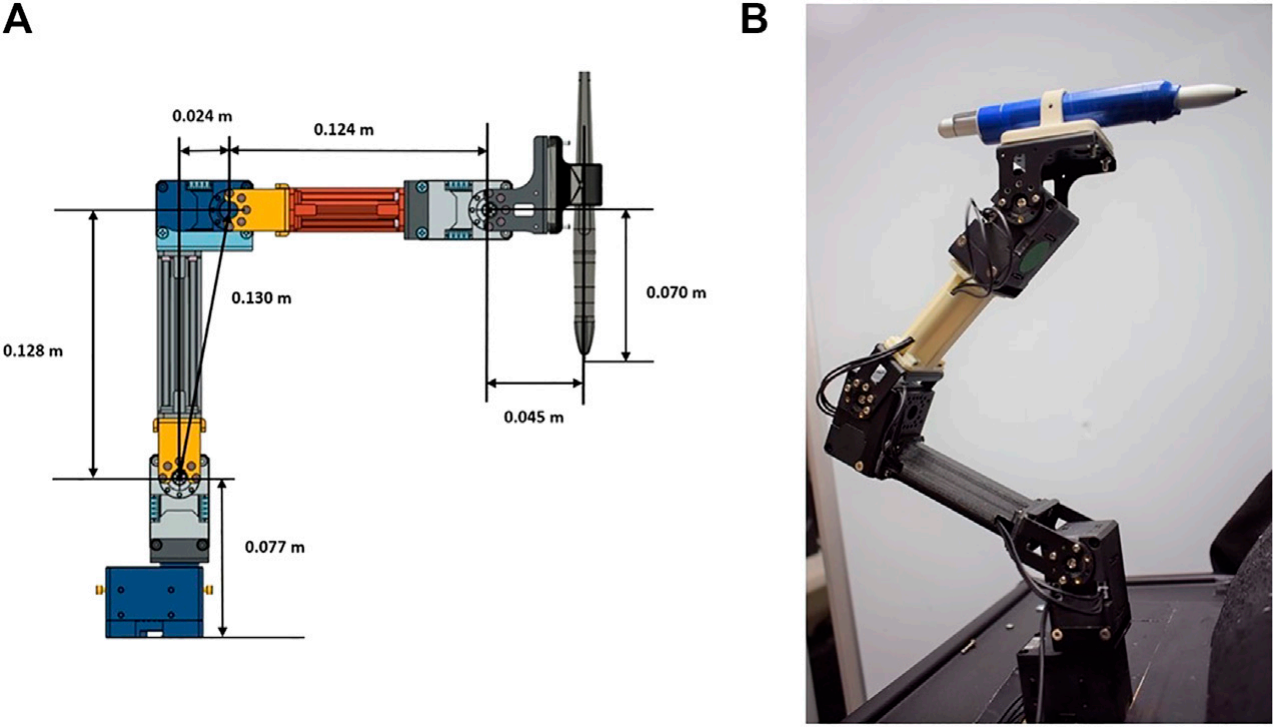
Robot manipulator prototype **(A)** prototype CAD model and **(B)** built prototype.

The robotic platform used in this study is a modified version of the Robotis OpenManipulator-X (Robotis, 2020). Based on the Robot Operating System (ROS) framework, this platform is open-source and open-hardware, i.e., its controllers and CAD models of most of its components are accessible and free to use (see Figure 4A). This robot platform’s system configuration is a four DoF arrangement, with a pen holder tool holding a stylus pen (see Figure 4B), which interacts with the ICPT during operation. For the controller to function correctly, its program has been altered to account for the modified end effector, the number of actuators used, and each link’s dimensions to accurately calculate the forward and inverse kinematics. The modified manipulator consists of four Dynamixel XL430-W250-T servomotors and two 3D-printed links made of polylactic acid (PLA) that are connected by means of metal brackets (see Figure 4A). The end effector is a PLA 3D-printed pen holder that holds the stylus pen to interact with the screen. The load capacity of the modified manipulator is conservatively estimated to be 160 g which can easily accommodate the 15 g end effector and 20 g stylus pen. A Raspberry Pi 4 (RPi4), with 4GB of RAM and with ROS Melodic installed on Raspbian-Buster OS, controls the robot manipulator using the ROS packages executing on it. Using this powerful and cost-effective single-board microcomputer gives the system sufficient capacity to control the robot and run computer vision algorithms without compromising the system’s memory. Its small dimensions also make it simple to install and locate it near the system without interfering with the robot manipulator workspace.

**FIGURE 5:**
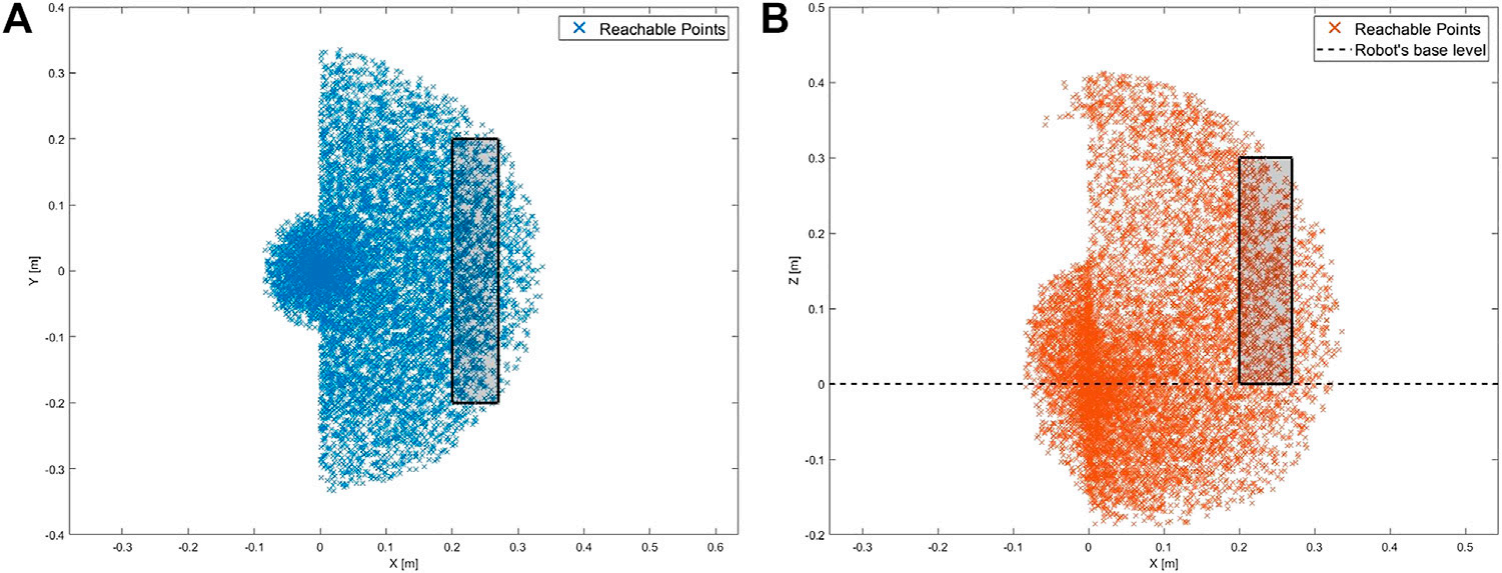
Workspace of the robot with the rectangular regions showing the range of allowable positions for the ICPT **(A)** top-view of the workspace and **(B)** side-view of the workspace.

To determine the workspace of the robot manipulator, the forward kinematics are first determined using the Denavit–Hartenberg (D–H) convention (Spong et al., 2006). Then, the Monte Carlo method is employed to generate the manipulator’s work envelope using the forward kinematics equations along with random sampling of permissible joint angles (Jianjun et al., 2018). This method produces a graphical representation of the manipulator workspace (Guan and Yokoi, 2006) that in turn is used to determine the range of ideal positions to install the robot relative to the medical device ICPT monitor. The allowable maximum and minimum distances between the robot and the medical device ICPT are determined to be 0.27 m and 0.20 m, respectively. The maximum distance is determined as the maximum distance between the robot and the ICPT that ensures that the entirety of the ICPT lies within the estimated workspace of the robot. The minimum distance is obtained by placing the ICPT as close to the robot as possible while ensuring that all of the interactions and the fiducial marker on the ICPT remain visible to the camera (see Figure 5).

To establish the achievable accuracy and repeatability of the robot, tests are conducted by commanding it to move the end effector from its home position of (*x* = 0.09, *y* = 0.0, *z* = 0.284) m to a test position and then returning the end effector back to its home position. This test is conducted for five test positions, one at each corner of the ICPT and one at the center, with the position of each test point measured relative to the lower left corner of the ICPT. Moreover, the process is repeated 50 times for each test point and the computed accuracy and repeatability are provided in Table 1. Note that the accuracy represents the distance between the desired test position and the average of the achieved positions. Moreover, the repeatability represents the radius of the smallest circle that encompasses all of the achieved positions corresponding to a desired test position (Mihelj et al., 2019).

**TABLE 1:** Robot accuracy and repeatability test results.

	*P*_1_ (*u*, *v*)	*P*_2_ (*u*, *v*)	*P*_3_ (*u*, *v*)	*P*_4_ (*u*, *v*)	*P*_5_ (*u*, *v*)
Ideal (mm)	(128.5,84.3)	(55.1,151.6)	(206,151)	(204.5,18.1)	(56,20)
Accuracy (mm)	0.55	0.06	0.21	0.19	1.00
Repeatability (mm)	1.29	1.13	0.88	1.76	1.03

### 2.2 USB Camera and Camera Calibration

The USB camera used in this setup is a C920 HD Pro Webcam, configured to capture a 640×480 image. By executing the camera driver on ROS, the webcam capture is made available as a ROS topic and becomes accessible to any subscribing program. Next, we perform a one-time geometric camera calibration using a pattern on a planar surface (Zhang, 2000), allowing the system to correct the image for lens distortion and to detect and measure objects in world units by determining camera location in the scene. These calibration parameters are estimated using an available ROS package for camera calibration and are stored as a file, to be later used during operation by the HRI interface and estimate spatial coordinates.

### 2.3 Communication with HRI Interface

**FIGURE 6:**
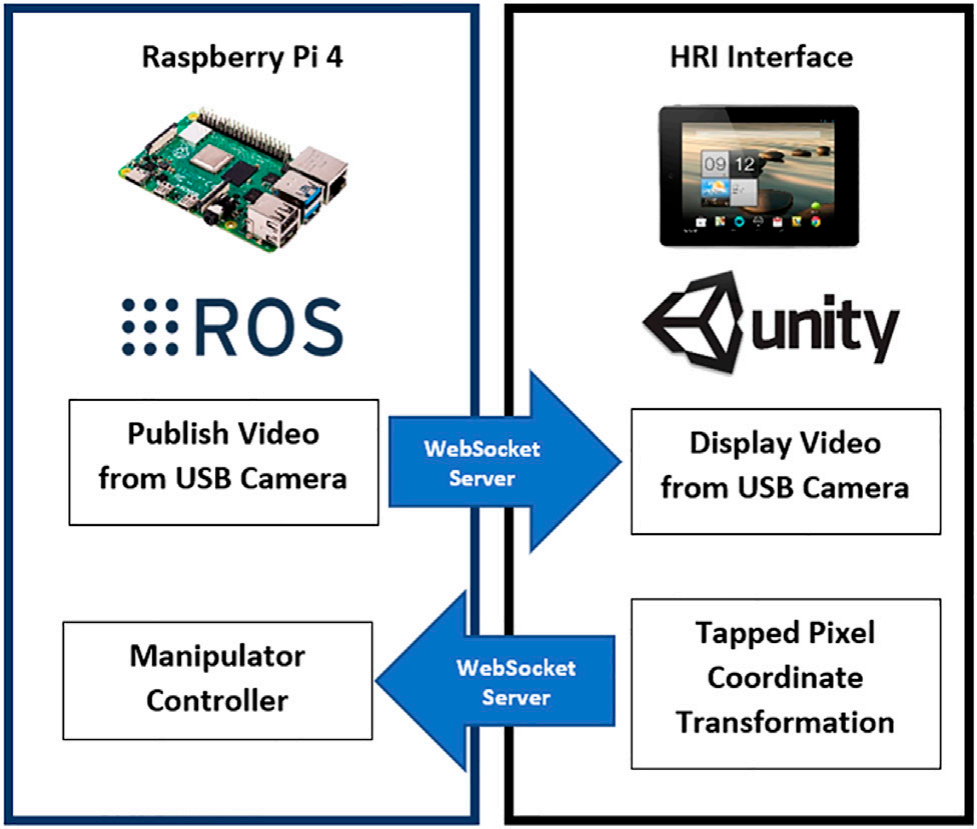
Communication between RPi4 and App.

Using the built-in Wi-Fi adapter of the RPi4, the information generated and published by the nodes running on ROS is made accessible to all members of the network on which the microcomputer is connected. Using a WebSocket server node on ROS establishes a communication bridge and allows web interaction with the ROS topics using an IP address and a port number. Upon joining as a client, the mobile App used for the HRI interface communicates with the RPi4 server and accesses the information running on ROS. This mobile HRI interface, developed using the Unity Engine (Unity Technologies, San Francisco, CA) and a freely available ROS asset, lets the App publish and subscribe to ROS topics (see Figure 6). When the application first starts on the mobile device, it immediately looks for the IP address and port to establish communication with the RPi4 microcomputer. The RPi4 and the mobile HRI interface are connected to an *ad hoc* wireless network created using a Netgear Nighthawk X10 AD7200 Wi-Fi router. For the laboratory environment of this study, the maximum range of the wireless network is experimentally obtained to be 27 m.

### 2.4 Reference Marker Detection

The approaches initially considered for the design of the HRI user interface in this work can be distinguished by the number of reference markers affixed on the medical device ICPT monitor, i.e., (1) four markers approach and (2) single marker approach.

#### 2.4.1 Four Markers Approach

**FIGURE 7:**
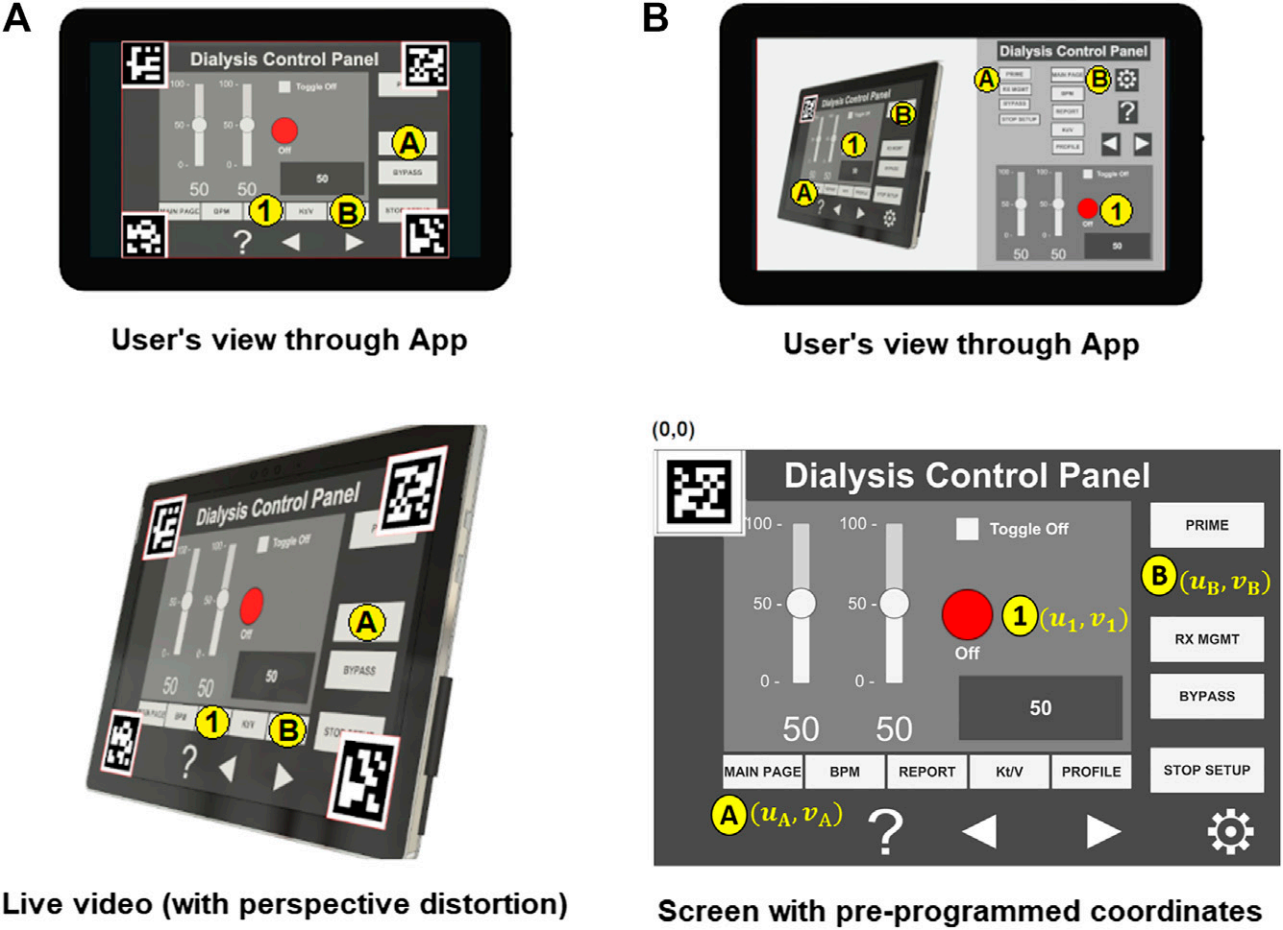
Reference marker detection **(A)** four marker approach and **(B)** single marker approach.

Using the projective transformation technique (Hartley and Zisserman, 2003), with four markers, allows the estimation of any location on the instrumentation control panel displayed in the video-feed on the touchscreen monitor. The four markers are placed on each corner of the ICPT monitor (see Figure 7A) and detected from the USB camera capture. The video-feed of the camera is used to estimate its real-world 3D pose (relative to the plane formed by the four markers) and subsequently to compute the pose of any point on the ICPT monitor relative to the camera’s coordinate frame. In this approach, the user can select each button of the ICPT by touching the corresponding location of the button on the streaming video image shown on the UI of the TCT (see Figure 7A, top panel). Moreover, the markers’ detected points are used to correct the perspective distortion caused by the placement of camera relative to the ICPT monitor and to scale the image to fit it on the UI of the TCT display. This method relies on two assumptions: (1) visual markers affixed to the ICPT monitor and interactive control elements (buttons and sliders) of the instrument control panel are on the same plane (coplanar points) and (2) the base location of robot relative to the camera position can be estimated (see subsection 2.5). Even though this approach can allow our system to interact with any medical machine with an ICPT, regardless of the ICPT function arrangements, placement of four markers on the same plane as the machine screen, in some cases, may block portions of the display containing important information for the machine functionality.

#### 2.4.2 Single Marker Approach

This approach uses only one reference marker (see Figure 7B). The system localizes the robot relative to the marker’s position using marker corners as correspondences to perform a projective transformation, but reducing the accuracy of the estimation (compared to the four markers approach) due to the lower number of correspondences detected. With this in mind, the robot control needs to be pre-programmed using the *a priori* knowledge about the locations of the on-screen control elements (buttons and sliders) relative to the attached marker to establish a one-to-one correspondence. For example, when the user touches button A on the UI of the TCT, the corresponding location (*u*_1_, *v*_1_) for the ICPT needs to be assigned automatically as the intended location. The UI executing on the TCT consists of a streaming video panel and a button panel. For each button on the medical device ICPT monitor, a corresponding button is available on the button panel of the UI on the TCT. This approach also assumes that the location of robot relative to the camera position can be estimated. However, requiring information about the arrangement of control elements on the ICPT to pre-program the UI of the TCT will limit the usability of this arrangement since the on-screen layouts of control panels may vary between machines of different manufacturers and specially for different medical machines. Moreover, not having the well-defined four corners of the surface plane of action (as in the four markers approach) limits the system’s ability to accurately correct perspective distortion (see Figure 7B, top panel).

#### 2.4.3 Hybrid Approach

**FIGURE 8:**
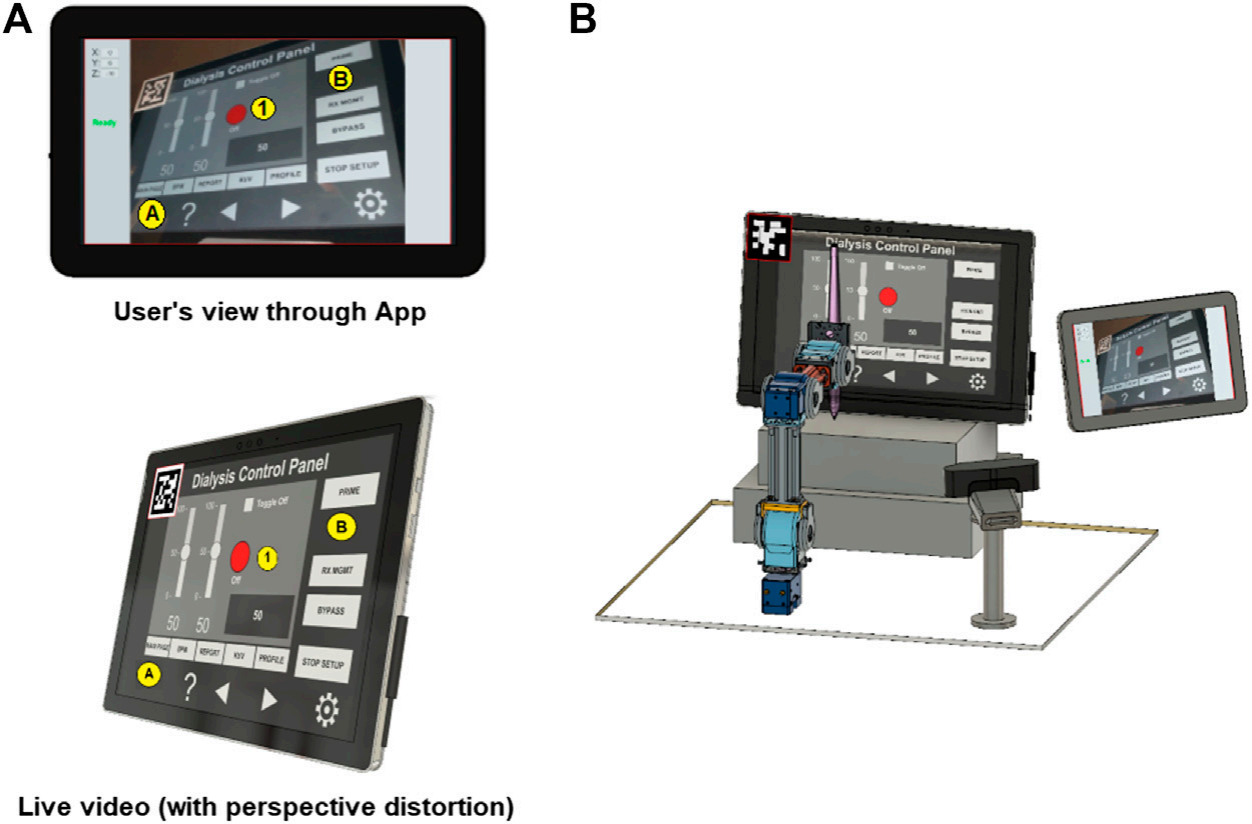
Reference marker detection **(A)** hybrid marker detection approach and **(B)** complete setup with hybrid approach.

In this paper, we present an early *proof-of-concept* that employs a hybrid approach by building on the two methods discussed above (see Figure 8). By subscribing to the image published by the camera driver node on ROS, the mobile App gains access to its video-feed that contains a single ArUco marker (Garrido-Jurado et al., 2016) placed on the top-left corner of the screen and detects it using the open-source ArUco module (Romero-Ramirez et al., 2018) of the Open Source Computer Vision Library (OpenCV). Instead of requiring a pre-programmed control panel on the UI of the TCT with known locations of the control elements on the ICPT (as in the single marker approach), the UI now detects the reference marker’s corners, and an algorithm estimates the homography (Corke, 2013) between the camera image to the surface plane of the reference marker. With this transformation and the information from the camera calibration file, the mobile App maps coordinates of a user-selected pixel on the video streamed image on the UI of the TCT to a spatial coordinate on the ICPT in world units, relative to the camera’s reference frame. As in the previous approaches, this approach assumes that the robot base location relative to the camera can be estimated. Its functionality is similar to the four markers approach, letting the user select a control element (button or slider) of the ICPT by touching its corresponding location on the UI’s image on the TCT. However, its accuracy may be compromised due to the limited number of correspondences detected.

As described above, the usability of the hybrid approach benefits the system by not relying on the *a priori* knowledge of arrangement of the control elements on medical device ICPT or on risking portions of the ICPT being blocked by the placement of multiple markers, however it has less accuracy than the four markers approach. The hybrid approach will also not correct the captured image’s perspective of the USB camera for the UI displayed on the TCT. In future research, we will test, compare, and contrast the usability and performance of the three approaches by conducting user tests to assess various parameters of UIs (such as intuitiveness, user-friendliness, perception, and remote operation workload) and the robotic device (such as accuracy and repeatability).

### 2.5 Camera Position and Robot Calibration

To allow the robot manipulator to interact with a point in its workspace (on ICPT) corresponding to any point selected by the user on the mobile App screen (on TCT), the robot controller requires the corresponding spatial coordinate specified in the robot’s frame of reference (located on the center of the robot base). This necessitates imparting the system knowledge about the camera’s pose relative to the robot frame of reference (^*R*^ *T*_*C*_). Thus, a calibration routine is created and implemented before the system starts any HRI operations. That is, this routine is run immediately after the camera’s orientation has been established to capture the robot’s workspace surface (i.e., the ICPT monitor).

**FIGURE 9:**
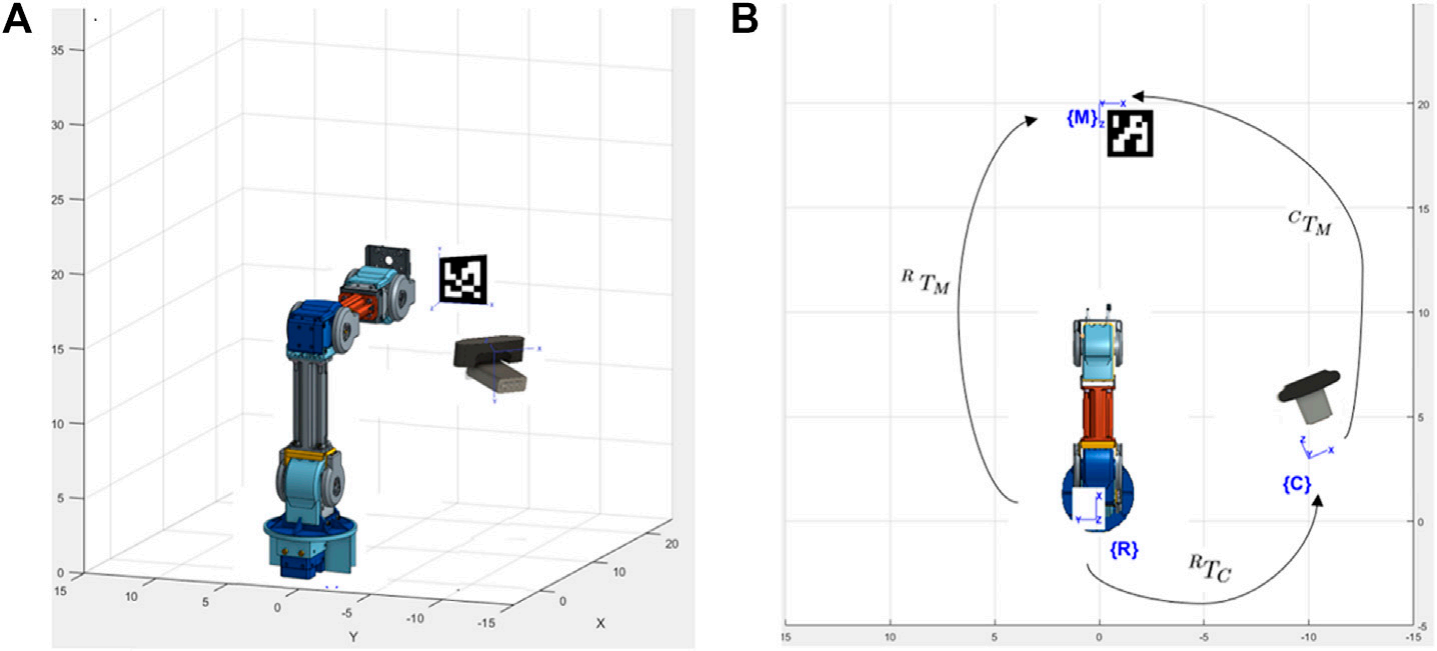
System setup to estimate camera pose **(A)** isometric view and **(B)** top view.

We first locate the ArUco marker in a predefined pose relative to the robot’s reference frame (see Figure 9). With this known pose (^*R*^ *T*_*M*_) and with the pose of the marker relative to the camera reference frame (^*C*^ *T*_*M*_), estimated by the mobile App, the calibration routine computes ^*R*^ *T*_*C*_ as follows

TRC=TRM(TCM)−1. (1)

Now ^*R*^ *T*_*C*_ is stored on and used by the mobile App to map pixel location of any point tapped by the user on the TCT to a spatial coordinate on the ICPT in the robot’s reference frame. To achieve a mapping from the TCT to ICPT of any size, the user enters, in millimeters, the width and height of the ICPT, and the *u* and *v* offsets of the top left corner of ICPT from the center of the fiducial marker, into the App. This creates an interactive region on the TCT that is the size of the ICPT as seen on the video-feed on the TCT. Next, to map any desired point on the ICPT to the robot’s workspace, we first locate the fiducial marker of known size (40 mm × 40 mm) on the ICPT surface. Based on the size and orientation of the marker obtained using computer vision, the App obtains the marker’s pose relative to the camera position. It uses this information to map any pixel coordinate to a space coordinate relative to the camera frame. Finally, using the transformation matrix (^*R*^ *T*_*C*_) obtained in the calibration step, the desired interaction point on the ICPT is mapped to spatial coordinates in the robot arm’s coordinate frame. This coordinate serves as the input to command the robot to move to the desired position. As long as the ICPT is located within the robot workspace and its entire screen (with the fiducial marker located on it) is visible to the camera, the robot can reach any desired point. Finally, once the App maps the TCT coordinate into a spatial coordinate, it is published to a ROS topic, making it available to the robot manipulator controller. However, even if the calculation of ^*R*^ *T*_*C*_ is accurate, there may be slight residual errors in the end effector’s final position. To compensate for this, the second part of the calibration routine consists of commanding the robot to go to the center of the reference marker multiple times. The user moves the end effector’s final position by tapping on the UI screen at preprogrammed buttons, which are displayed during the calibration routine, to manipulate the stylus pen’s tip in the *X*, *Y*, and *Z* directions until it matches the marker’s center as precisely as possible. The offset values needed to reach the actual desired position are stored and used to increase accuracy during the operation.

### 2.6 Robot Operation

A program on the RPi4 runs a ROS node that uses the information from the mobile App and uses the controller node of the manipulator robot to move it to the user-specified location. After performing the calibration routines, the system is ready to operate. The manipulator robot control program moves the robot to an initial position and waits for a user-specified coordinate to be available on the ROS topic where the mobile App publishes coordinates.

**FIGURE 10:**
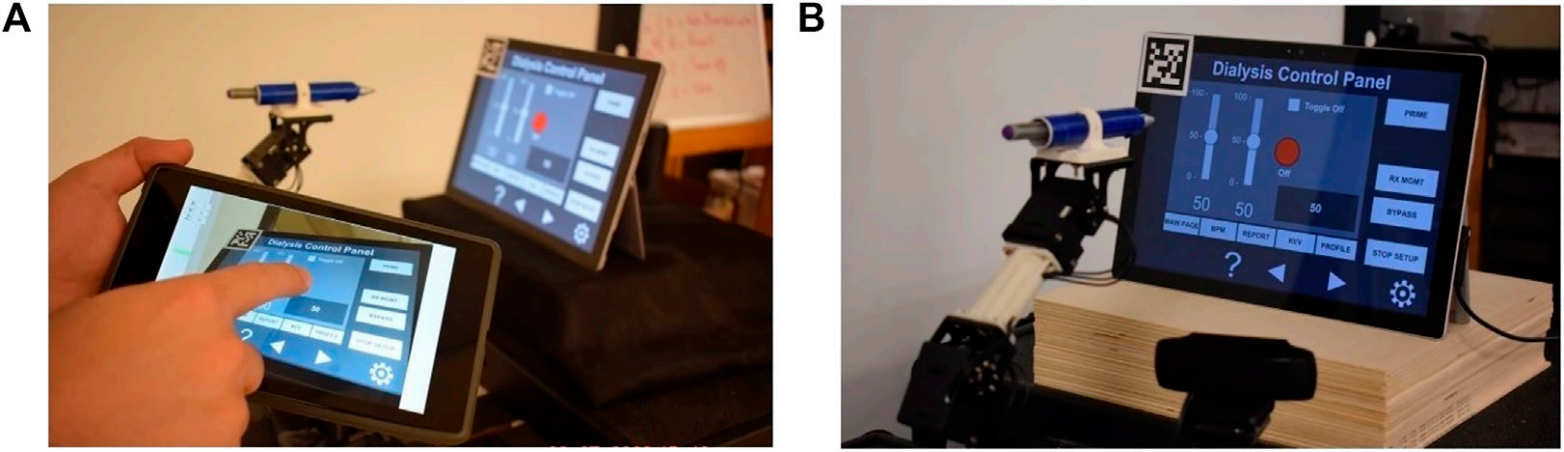
**(A)** HRI interface and **(B)** complete robot system setup.

When the App starts, it immediately tries to communicate with the microcomputer. Once the communication is established, the touchscreen of the tablet device running the App will show the streaming video from the camera located next to the robot, capturing the images from the ICPT (see Figure 10A). With the detected 2D reference marker’s information, the App will wait for the user to tap on the display of the TCT. The moment a new user-specified coordinate is received, using a sequence of events, the robot control program: (a) moves the robot manipulator to the desired location, just over the specified coordinate on the surface plane of the ICPT; (b) performs a tapping action that consists of moving slightly toward the ICPT until a contact occurs; and (c) returns to the initial position and waits for any new coordinates to be made available. This robot control program reads and responds to only one user-specified coordinate at a time and ignores any newly sent user coordinates while performing the sequence of operation for a previously received coordinate.

The complete system setup created for this proof-of-concept (see Figure 10B) uses a Microsoft Surface Pro 4 computer as the ICPT, running an application that mimics the functions of a dialysis machine instrument control panel.

## 3 System Evaluation

An experimental study was conducted with participants to evaluate the performance and usability of the proposed system. The study was conducted with two groups of users, referred hereafter as the in-person and remote groups. In the in-person group, 17 participants performed the experiment in a room adjacent to the room housing the robot, camera, and ICPT monitor. Alternatively, in the remote group, 16 participants performed the experiment from a remote location via the internet. See http://engineering.nyu.edu/mechatronics/videos/mhrifordialysis.html for a video illustrating a user interacting with the prototype to complete a set of tasks. Prior to performing the experiment, participants in both groups were briefed individually on the purpose of the experiment, what it entails, and how do the interactions take place. They were informed that when the “Ready” prompt is shown on the TCT, the user can issue a command to the robot and when the “Busy” prompt is shown, it means that the robot is executing a task and will not accept any user command until the task is completed. No pretrial was conducted and each participant performed the experiment for only one time. This was done to ensure that the participants did not have any prior knowledge about the capabilities and the overall responsiveness of the system.

The participants who performed the experiment in-person were asked to use an Android tablet device with a touchscreen and interact with its screen using a stylus. During the experiment, the tablet device was connected to the same dedicated wireless network that the robot was connected to, and each user performed the experiment by staying in the same location in the room.

To test whether controlling the robot from a remote location has any influence over the system usability, system performance, and the task load of the user, an online study was conducted wherein the participants were asked to command the robot by assuming control of a computer connected to the dedicated wireless network shared by the robot. The participants were briefed in a similar manner to those in the in-person experiment, and no pretrial was conducted for this group either. The only major difference between the two groups was that the remote group of participants were interacting with the video-feed using a mouse pointer on their computer, whereas participants in the in-person group were interacting with a tablet device using a stylus to issue commands to the robot.

**FIGURE 11:**
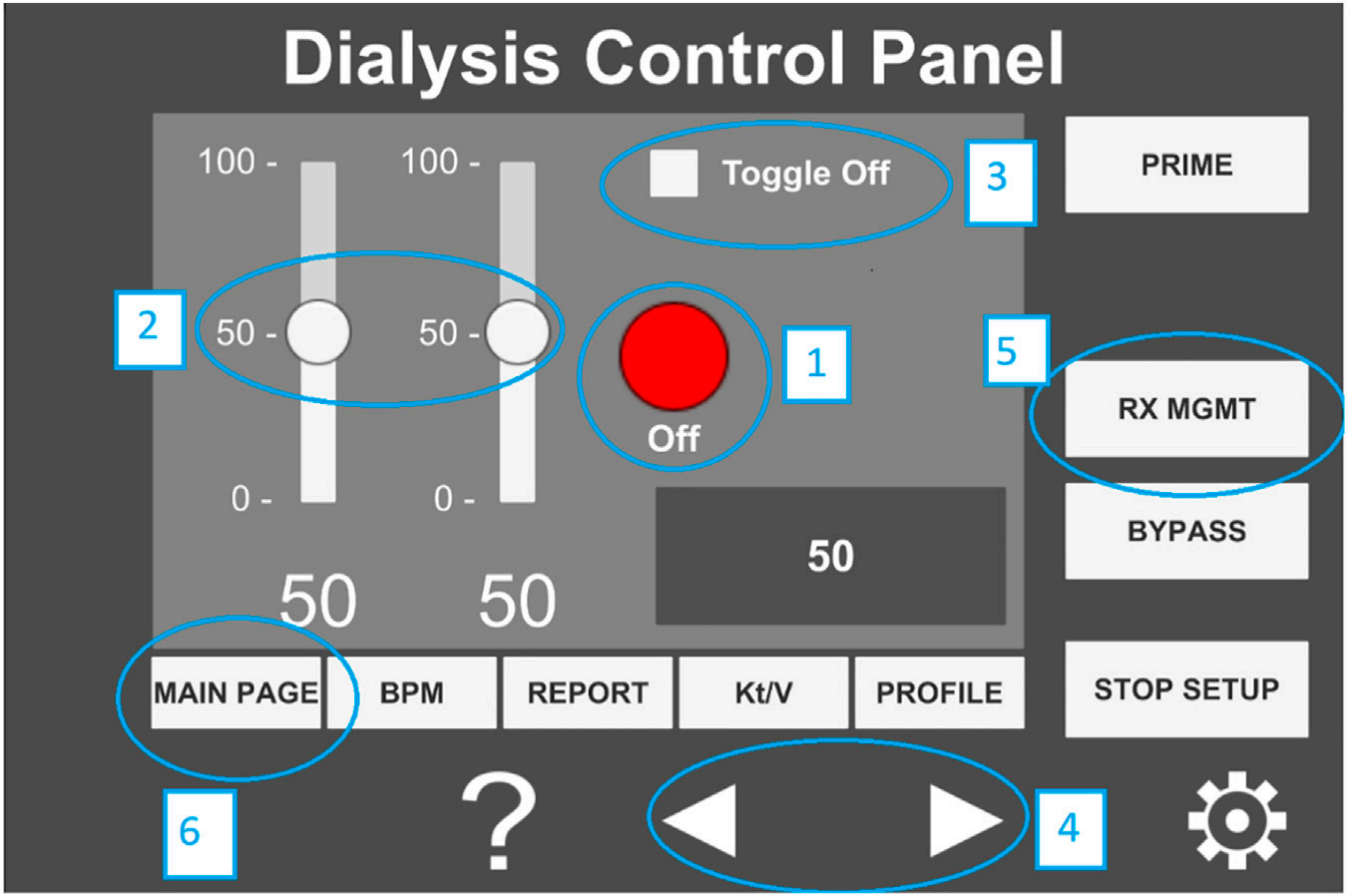
Annotated image of the user interface hosted on the tablet computer.

During the experiment, the participants were asked to read a set of instructions on a PDF document and perform the experiment accordingly. The PDF instruction document listed six numbered tasks and an accompanying annotated image of the user interface (see Figure 11), where the six tasks correspond to six different interactions that the users needed to perform. These tasks were designed to mimic a set of user interactions that a healthcare worker typically performs on a dialysis machine interface. The details of the interactions are as follows.

(1)Press the red ON/OFF button.
(2)Change the value of the left slider to ‘0’ and the value of the right slider to ‘100’.
(3)Press the toggle button.
(4)Increase/decrease the value displayed in the gray box using the arrow buttons.
(5)Select the RX MGMT button.
(6)Return to the main display using the MAIN PAGE button.

After the participants performed the six tasks, they were asked to respond to two questionnaires that assessed their experience for qualitative evaluation. The first part of the evaluation required the participant to respond to the NASA-Task Load indeX (NASA-TLX) (Hart, 2006) to assess the workload experienced by the participants while using the system. The NASA-TLX is used to rate the perceived workload of an individual while performing a task. It is divided into six categories that include physical workload, mental workload, temporal workload, effort, frustration, and performance. In this study, the Raw TLX (RTLX) assessment was performed in which the TLX scores are unweighted and the overall load of the task is calculated as the average score of the six categories in the NASA-TLX. In the second part of the evaluation, the participants were asked to express their level of agreement on a System Usability Scale (SUS) (Brooke, 1996) questionnaire. The questionnaire consists of the following five positive and five negative statements with responses on a 5-point scale (1: strongly agree and 5: strongly disagree).

(1)I think that I would like to use this system frequently.
(2)I found the system unnecessarily complex.
(3)I thought the system was easy to use.
(4)I think that I would need the support of a technical person to be able to use this system.
(5)I found the various functions in this system to be well integrated.
(6)I thought there was too much inconsistency in this system.
(7)I imagine that most people would learn to use this system very quickly.
(8)I found the system to be very cumbersome to use.
(9)I felt very confident using the system.
(10)I needed to learn a lot of things before I could get going with this system.

The participants were provided Uniform Resource Locators (URL) to the NASA-RTLX and the SUS questionnaires and were asked to complete them on the spot immediately after completing the six-step interactive tasks provided above. The questionnaires were kept anonymous and no personal information was asked from the participants except their age group and their gender.

## 4 Results

The performance and the user experience of the proposed system was evaluated by conducting a study with 33 participants, of whom 29 participants were either engineering students or professionals working in a STEM related field and the remaining four were medical professionals. A majority of the participants (72.73%) had operated or programmed a robotic system while the rest 27.27% had neither operated nor programmed a robot prior to their participation in the study. Note that the four medical professionals were part of the remote group and only one of them reported to have programmed or operated a robotic system previously. Furthermore, qualitative data obtained from the SUS questionnaire contained two outliers and one participant from the remote group did not complete the NASA-RTLX self assessment. Thus, the data obtained from these three participants was not used for system evaluation and a total of 30 participants’ data, 15 from each group, was used for the results reported below.

### 4.1 System Performance

First, the performance of the system was evaluated by validating the accuracy with which a user is able to select and interact with desired points on the ICPT monitor using the proposed HRI interface on the TCT. We considered five reference points on the ICPT. These points were located at the center (*P*_1_(*u*, *v*)) and near the four corners (*P*_*i*_(*u*, *v*), *i* = 2, . . .,5) of the screen. The experiment was conducted 50 times by a single user for each of the five reference points. The user input when interacting with TCT was recorded as pixel coordinates along the *u* and *v* axes and referred to as the *commanded* value. The point at which the robot interacted with the ICPT in response to the commanded value is referred to as the *measured* value and it was also stored as pixel coordinates along the *u* and *v* axes. The pixel coordinates for the commanded values were scaled up to the screen resolution of the ICPT so that a direct comparison with the measured values could be made. The performance of the HRI interface was evaluated by calculating the absolute difference between the commanded and measured values for each interaction. Then the average absolute error was calculated for both the *u* and *v* coordinates. This was done for all five reference points and the results are shown in Table 2. The results indicate that for all five reference points, the highest average absolute error was less than 18.54 pixels for the *u* coordinate and 26.98 pixels for the *v* coordinate. Given that the resolution of the screen used for the ICPT is 2,736 × 1,824, with a diagonal screen size of 12.3 inches (312.42 mm), the pixel-to-length ratio was found to be 10.5 pixels/mm. Thus, the maximum average absolute error was 2.56 mm in the *v* coordinate of the fifth reference point *P*_5_(*u*, *v*). It is important to note that there was a button located at each of the five reference points and all 50 tests conducted on each button were successful, i.e., the button was successfully pressed each time. The diameter of the buttons is 90 pixels which is approximately equal to 8.6 mm. This particular size of buttons is chosen because it is considerably smaller than all interactive elements on the touchscreen and the touch pad of a dialysis machine, and therefore proves to be a reliable indicator of the performance of the system.

**TABLE 2:** Performance test results.

Values in pixels	*P*_1_ (*u*, *v*)	*P*_2_ (*u*, *v*)	*P*_3_ (*u*, *v*)	*P*_4_ (*u*, *v*)	*P*_5_ (*u*, *v*)
Ideal	(1368,912)	(568,1612)	(2168,1612)	(2168,212)	(568,212)
Commanded (average)	(1367.4,912.1)	(569.7,1601.6)	(2166.9,1612.1)	(2163.2,211.4)	(595.9,215.9)
Measured (average)	(1385.9,905.7)	(565.8,1596.9)	(2162.6,1598.0)	(2173.6,215.9)	(599.8,189.1)

The time taken by the robot to complete an interaction is determined by the task time programmed for the robot. In experimentation, it is measured as the difference between the time when the robot receives a command and the time when the robot returns to its home position after performing the interaction. The robot took 12.036 s to complete an interaction, without any significant difference in the times spent for different interactions. Next, the time it takes for a user to complete an interaction on the *ad hoc* wireless network is calculated as the difference between the time when the command is sent by the TCT and the time when the user receives the “Ready” prompt again on the TCT. For each of the following three scenarios, 15 tests were performed to measure the user interaction completion time.

(1)The user holding the TCT and the robot are in the same room.
(2)The user holding the TCT and the robot are in different but adjacent rooms.
(3)The user holding the TCT is at the maximum working distance from the wireless router (27 m), with multiple rooms in between the user and the robot.

In all three scenarios, the average time to complete the user interaction showed no significant difference and was found to be 12.077 s. Finally, in the last time measurement experiment, we sought to determine the user interaction time when performing interactions with the robot over the internet. With a user located at a distance of approximately 1.5 mi from the robot, the average interaction time for 15 tests was obtained to be 12.56 s. Note that while the task completion time for the robot remains constant, the task completion time for the user and the maximum allowable interaction distance from the robot can change depending on user location and Wi-Fi signal strength, respectively.

### 4.2 User Experience

While the results obtained using the system performance test validated the utility of the proposed system from an accuracy and precision point of view, it is important to consider the overall user experience when the participants operate the system. Thus, three different methods were used to perform the qualitative analysis of the user experience. The first method involved measuring the task load of the experiment using the NASA-RTLX self-assessment. This was followed by administering a system usability questionnaire, and finally the verbal/written feedback given by the participants was reviewed.

#### 4.2.1 Workload

The assessment of the workload was performed by analyzing the results obtained from the NASA-RTLX self-assessment for the in-person and the remote participant groups. All categories were scored on a scale of 0–100 and the overall score for each participant was computed as a mean of the score for the six categories.

**FIGURE 12:**
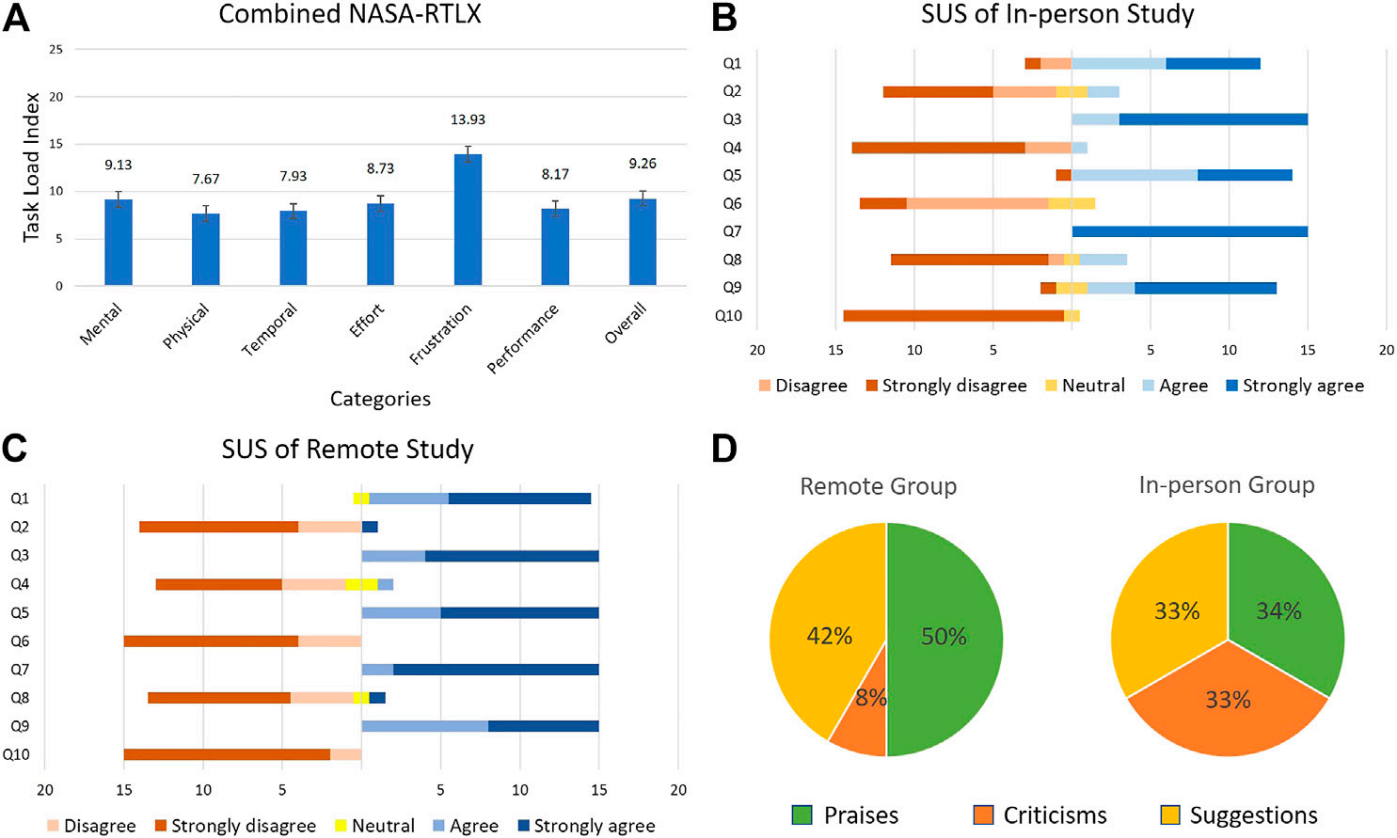
User study results **(A)** NASA-RTLX **(B)** SUS in-person study **(C)** SUS remote study, and **(D)** user opinion.

The score for each category was averaged and these calculations were used to compute the mean overall workload for both groups. Since there was no overlap between both groups, and therefore both samples are independent, a Welch’s unequal variances two-tailed *t*-test was performed on the individual categories of the NASA-RTLX scores from both groups and the tests yielded *p* > 0.05 for responses of in-person *vs.* remote experimenters. Thus, it was concluded that there was no statistically significant difference between the task loads experienced by the participants in the two groups. The combined average task load for both groups is computed and reported in Figure 12A.

The collective task load values for two groups (Figure 12A) and the raw data indicate that the frustration score was the highest for the two groups. The high frustration value can be attributed to the downtime that the participants experienced during the “Busy” phase of the robot movement, when the participants could not issue new commands to the robot. When we consider this factor with the slow speed at which the robot moves, it is plausible that the frustration value would increase as a result. Upon further examination of the raw task load data, it was observed that among the six categories, the effort scores exhibited a relatively high inter-group difference (in-person effort = 11.67, remote effort = 5.8). The difference between the effort values of both groups can be explained as a result of the type of interaction method with the robot. Participants in the remote group issued commands to the robot via a mouse pointer on a computer. This gave them very fine control with pixel perfect accuracy and a large screen size that definitely helped in the experiment. On the other hand, participants in the in-person group were asked to use a tablet device and a stylus to interact with the video-feed. The stylus requires extra pressure to be applied on the tablet computer screen to register a touch input and the smaller screen size required the users to pay more attention to where they were interacting with the screen of the tablet device.

#### 4.2.2 System Usability

To gain an insight into the user experience of the participants, they were asked to complete a system usability questionnaire using a 5-point scale (1: strongly agree and 5: strongly disagree). The participants’ individual responses were subjected to an unequal variances two-tailed *t*-test and the responses for the in-person group were compared with those of the remote group. Out of the 10 questions on the SUS questionnaire, three questions [(1), (5), and (6)] showed a statistically significant difference with *p* = 0.03, *p* = 0.03, and *p* = 0.001, respectively. Upon close examination of the data, two in-person group participants’ responses were identified as outliers due to the large distance between their responses and the mean response for questions [(1), (3), (5), (7), and (9)]. Upon removing the outliers from the data, an unequal variances two-tailed *t*-test was performed again on the responses from the remaining 15 participants in the in-person and 15 participants in the remote groups. The results are shown in Figures 12B,C.

Out of the 10 questions, only question (6) showed a statistically significant difference with *p* = 0.001. Although all participants in the remote group disagreed or strongly disagreed that there was too much inconsistency in the system, some participants from the in-person group had neutral responses on this question. The neutral responses can be interpreted as participant reservations on the responsiveness of the tablet device when interacting with it using a stylus. Since the stylus used in this experiment had a relatively large tip, it is possible that some participants found inconsistencies when interacting with the tablet device if they did not pay close attention to where they touched the screen. This also explains why the participants in the remote group did not find any inconsistencies despite controlling the robot from a remote location. Since remote participants were using a mouse pointer on a comparatively larger screen (using a laptop or desktop computer), they could direct the robot more precisely, therefore reducing the human input error. A viable solution that would alleviate the problems faced by the in-person group would be to use a tablet device with a larger screen size, and/or use a stylus with a finer tip.

### 4.3 User Comments

From the participants who tested the prototype, remotely and locally, we obtained different insights about their experiences interacting with the system by reviewing their comments and feedback. A total of 33 individuals participated in this study, out of which 21 provided comments and suggestions about their experience in controlling the manipulator. Some of the comments praised the system as evidenced by the use of terms such as “helpful,” “efficient,” “easy-to-use,” “pretty good,” “requires very little experience,” among others. Although several other participants did not express negatively biased comments, they expressed some reservation with the speed of the robot in executing the received commands, e.g., “the time taken by the robot to execute the command slows the process down.” There were also criticisms from users who tested the prototype from a remote computer and on-site with a mobile device regarding the smoothness of the robot movements and the camera image shown in the HRI interface. Specifically, a participant who tested the prototype in-person using a tablet, suggested making the robot “more robust” and another participant who used the robot from a remote computer, advised “make the system more accurate and more stable […] decrease the skew in the image from the camera.”

Since this study proposes a solution to be used by heathcare workers, we also reached out to doctors who were willing to test the prototype and provide a review based on their experience working during the COVID-19 emergency. A total of four doctors remotely interacted with the proposed system and provided their feedback which included suggestions, criticisms, and compliments about the system and its utility as a viable solution for the control of dialysis machines during a pandemic. For example, one of the participant doctors, who used the prototype from a computer outside the United States, praised the system by commenting on its ease of understanding, use, sensitivity, absence of errors, ability to avoid contact with patient, etc. Another participant doctor offered insight into how this solution is perceived from a medical perspective, i.e., “interesting” and “of enormous use, especially when necessary to avoid physical contact.” He also advised to improve the precision of robot because sometimes “it was necessary to select the same task until it was completed successfully.” Additionally, another medical professional expressed his interest in how this system would perform in a real situation. This doctor provided a verbal review by stating that the system works very well but it will require testing on a real dialysis machine to see how it controls it.

We found that the difference in these reviewer experiences is partially explained by the variations of internet connection speeds available on each participant’s respective location (when controlling the system from a remote computer, off-site). In some cases, this variable added delay to video streaming, which did not let the users monitor how the robot was performing the tasks.

Figure 12D presents the percentages of each type of comments provided by the participants using the proposed system. We categorized the comments into “praise”, “suggestion”, and “criticism” categories. Positively biased comments were categorized as praises and accounted for 34% of the commenters from the in-person group and 50% of the commenters from the remote group. We categorized as criticisms the comments that identified a shortcoming without providing a recommendation. These accounted for 33% of the commenters from the in-person group and 8% of the commenters from the remote group. Finally, comments that provided recommendations to improve the system were categorized as suggestions and accounted for 33% of the commenters from the in-person group and 42% of the commenters from the remote group. It is seen that most participants were affected enough by their participation in the study to leave meaningful comments. Moreover, a sizable portion of participants was satisfied enough to praise their experience in writing. We took these praises to confirm our arguments for integrating mobile hardware and software as an effective way to interact with medical machines remotely using robot systems, such as the one proposed in this study. Many of the praises expressed the satisfaction of completing a set of tasks remotely, either using a “click” on a computer screen or tapping a location on a mobile device screen.

On the other hand, criticisms gave us areas of opportunity on which we can focus to improve our prototype to deliver greater satisfaction in the use of HRI interfaces to control robots remotely and to meet the expectations of the system performance while executing a task. Observations regarding the smoothness of the robot movements and precision allow us to understand better how a system of this nature is perceived. Even if the users complete a set of tasks successfully, the speed while performing this task or lack of smoothness on the manipulator movements creates some distress. On the other hand, criticism about the skew of the camera view confirms that there is also some level of discomfort when a user perceives a distorted perspective of a surface (touch screen) with which interaction is required. While many of these suggestions for improvements will be considered in developing and testing future prototypes, below we offer one improvement to render a distortion-free perception of the ICPT on the TCT.

### 4.4 Suggested Improvement Based on User Tests

**FIGURE 13:**
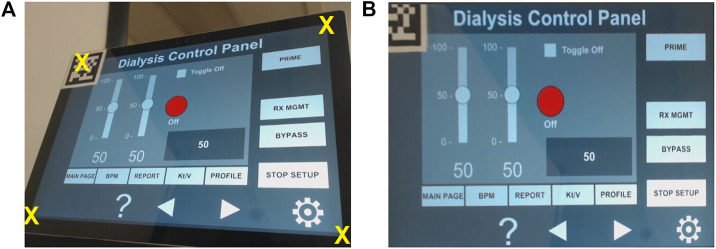
**(A)** Raw image with corners selected by the user and **(B)** image with distortion corrected.

On the SUS questionnaire and in the comment section, several participants provided written (and verbal) feedback concerning the skewed perception of the camera video-feed. In response, we have explored the potential of including an additional feature to the hybrid approach of this study, which uses a single reference marker, to correct and improve the video-feed displayed on the TCT interface. Specifically, as previously, when the users run the mobile UI App on the tablet device, the raw live-stream of the ICPT is displayed on the TCT with a distorted perception. Next, the App prompts the user to touch (from the mobile device) or click (from the remote computer) the four corners on the video-feed of the surface plane of action of the ICPT in a clockwise manner, starting from the corner closest to the fiducial marker (see Figure 13A). These user-selected pixel coordinates, corresponding to the corners of the ICPT, are used to get a perspective transformation matrix and map the identified ICPT plane to fit the screen of the TCT by performing a perspective correction. This correction technique allows the user to be presented with a distortion-corrected view of the raw ICPT video-feed in the HRI interface (see Figure 13B). When the user interacts with the corrected image displayed on the TCT, the inverse of the perspective transformation matrix computed above can be used to map the pixel coordinates of the user interaction on the TCT to the original perspective view captured by the camera, allowing the application to work without any additional modifications. Figure 13 illustrates that it is feasible to implement such a perspective correction approach, however a complete set of user-tests with this improved approach is beyond the scope of this study and will be considered in a broader study with the two alternative approaches suggested in subsection 2.4.

## 5 Conclusion

In this paper, we proposed a system for remote control of a dialysis machine with mobile HRI as part of COVID-19 emergency response. The proposed approach utilizes the capabilities of a smartphone/tablet device as a mode of interaction with a 4-DOF robot and explores the possibility of manipulating the robot to remotely interact with the instrument panel of a dialysis machine. This allows the medical professionals to maintain social distancing when treating dialysis patients, preventing potential exposure to pathogens for both the healthcare staff and the patients. Such a system will also help lower the use of PPE by doctors and nurses while performing routine, simple procedures that could be performed by a robot. To evaluate the proposed system, its performance, and the user experience, a user study was conducted in which participants remotely issued commands to a robot via a tablet device or a computer. The participants received a live streaming video of a mock dialysis machine ICPT that allowed them to command the robot to manipulate the UI elements of the ICPT by touching those elements on the video-feed on the TCT. Results of the study show that the participants were able to remotely access the UI elements of the ICPT and complete the tasks successfully. Based on the feedback received on the SUS questionnaire from the participants, an improvement to the proposed HRI interface was suggested and implemented which corrected the perspective distortion of the live-stream of ICPT and allowed the user to interact with the corrected image for a more intuitive exprience. Overall, the live streaming video of the instrument panel provides a very natural and intuitive mode of interaction for the user and does not require prior experience in programming or operating robots. Most importantly, there is no need to develop a custom UI for the TCT since the user directly interacts with the video-feed from the ICPT. This allows the proposed system to work with any touchscreen and the development of custom TCT interfaces that only work with their corresponding ICPT is not required. Finally, the proposed approach can be deployed very rapidly and requires minimum preparation work in case of an emergency, therefore saving valuable time and resources that can be directed elsewhere. Future work will incorporate force feedback control on the robot end effector and multiple fiducial markers on the ICPT to increase the accuracy of the robot. Furthermore, the possibility of a mobile robot platform will be explored, which will allow the user to interact remotely with multiple medical equipment in a given environment. Finally, additional intuitive modes of interaction involving wearable technologies and AR will be explored to enhance the user experience and user efficiency while minimizing the task load.

## Data Availability

The raw data supporting the conclusions of this article will be made available by the authors, without undue reservation.
